# A Simple Preparation of Crosslinked, Highly Alkaline Diallyldimethylammonium Hydroxide Hydrogel Particles via Inverse Static Anion Exchange

**DOI:** 10.3390/gels10110743

**Published:** 2024-11-15

**Authors:** Tim B. Mrohs, Oliver Weichold

**Affiliations:** Institute of Building Materials Research, RWTH Aachen University, Schinkelstraße 3, 52062 Aachen, Germany; mrohs@ibac.rwth-aachen.de

**Keywords:** hydrogel, ion exchange, particles, copolymer, DADMAOH, crosslinker

## Abstract

Highly alkaline hydrogels are gaining increasing attention in building materials research. Specifically, cationic alkaline hydrogels based on diallyldimethylammonium hydroxide (DADMAOH) as the monomer have been effectively used to seal water-bearing cracks or serve as coupling media for electrochemical chloride extraction. However, the residual halogen content and challenges in scaling up monomer production have hindered broader application. Attempts to use a commercially available cation-selective membrane for ion exchange achieved up to 90% chloride-to-hydroxide switch, but the approach proved ineffective due to significant monomer decomposition during the process. By contrast, neutral gels and gel particles can be readily prepared from diallyldimethylammonium chloride (DADMAC) in large quantities and with a wide range of compositions. It is demonstrated here that these neutral gel particles undergo inverse static anion exchange when suspended in NaOH solution, generating DADMAOH particles with residual halide contents of <0.3%, without the need for ion-selective or dialysis membranes. This corresponds to an up to 100-fold reduction in residual chloride content compared to particles produced directly from alkaline monomer solutions, thereby significantly enhancing the efficiency of hydroxide ion release. The swelling behaviour of the particles is primarily influenced by the initial monomer concentration, while conductivity remains largely unaffected, indicating that charge transport occurs mainly along the particle surface. Despite the pronounced increase in swelling with decreasing particle radii, the specific conductivity of 2.8 Ω^−1^ m^−1^ is still sufficient for their use as coupling media in concrete applications. In summary, the alkaline particles prepared via inverse static anion exchange meet all necessary requirements for building materials applications, offering a broader range of tuneable properties and greater ease of production compared to gels or particles derived from DADMAOH.

## 1. Introduction

Ionic hydrogels are crosslinked polymer materials with highly hydrophilic polymer chains that can absorb large amounts of water without dissolving. The bound water can either be freely present in the interstices of the polymer network or be tightly bound around the charges [1,2]. Hydrogels can absorb and release between ten percent and a thousand times their dry weight in water without losing their three-dimensional structure [3]. Hydrogels are typically synthesized from hydrophilic natural or synthetic polymers using either chemical or physical crosslinking methods [4,5]. In recent years, stimuli-responsive hydrogels, also known as “smart hydrogels”, have emerged as one of the most promising areas in hydrogel research. These hydrogels can alter their physical or chemical properties in response to external stimuli, such as temperature, pH, light, or magnetic fields [6]. A notable example of temperature-sensitive hydrogels is the composite poly (N-isopropyl-acrylamide) (PNIPAM) hydrogel combined with gold nanorods (AuNRs), as described by Zhang et al. These hydrogels demonstrate rapid and precise thermal responsiveness, with the integration of AuNRs enabling a swift increase in temperature, which in turn triggers controlled drug release [7]. Another significant advancement is the use of 3D printing technologies to create complex hydrogel structures that serve as scaffolds for tissue growth. These 3D-printed hydrogels allow the precise control of the structure and composition of the material, opening new possibilities in regenerative medicine and tissue engineering. Advances in bioprinting have enabled the embedding of cells directly into the hydrogel matrix, creating functional tissues that can potentially be used for transplantation or disease modeling [8]. Beyond biomedical applications, hydrogels have also found a place in environmental engineering. They are used for water purification, particularly for removing heavy metals or organic pollutants from wastewater. Due to their high water retention capacity and ability to incorporate functional groups, hydrogels can effectively act as filtration materials, binding and removing contaminants; these aspects underscore their sustainability and effectiveness in this field [9]. In the field of bioelectronics, conductive hydrogels have made significant progress. These materials have been successfully integrated into wearable electronic devices and biosensors. With their high flexibility and conductivity, they can transmit signals, serving as an interface between biological systems and electronic devices. Examples include flexible sensors that monitor bodily functions or electronic skin that can detect touch and movement [10,11].

A crucial aspect of hydrogel development involves advances in crosslinking methods. The way polymer chains are crosslinked significantly determines the stability, elasticity, and mechanical properties of the final product. Novel chemical and physical crosslinking approaches have allowed the creation of hydrogels that are more robust and versatile. The introduction of reversible crosslinking, for instance, has enhanced the self-healing capabilities of hydrogels, making them suitable for long-term applications [12].

For most applications, the gels are formulated in granular form, i.e., in the form of small discrete particles. A distinction is usually made between macrogels and microgels, where the latter are characterized by particle radii in the colloidal dimension, i.e., <1 µm [13]. Such ionic hydrogel particles can be produced in two different ways. The first is a bottom-up synthesis, in which microgel particles with very defined diameters can be synthesized from a monomer solution, e.g., by emulsion polymerization or microfluidics [14]. The other variant is top-down synthesis, in which a bulk macrogel is synthesized and then shredded into small fragments [15]. On an industrial scale, the top-down approach is preferred due to its suitability for larger batch sizes and its cost-efficiency. Additionally, unlike emulsion polymerization, this method does not require the subsequent separation of solvent mixtures. It is commonly employed for the production of poly-acrylate superabsorbents, which are used in baby diapers [16] and can be added to cement paste to mitigate autogenous shrinkage during hardening [17]. In the construction sector, the development of cationic hydrogels based on diallyldimethylammonium (DADMA^+^) has opened up new possibilities. These hydrogels can absorb and release mobile anions, with a particular emphasis on hydroxide ions, which is beneficial for maintaining the high alkalinity of concrete. They have been used as stationary ion exchangers to realkalize the surfaces of carbonated mortar and have proven effective in sealing water-bearing cracks [18]. Furthermore, they enable the coupling of electrodes to concrete surfaces during electrochemical chloride extraction (ECE), thereby improving the efficiency of these processes. The latter makes use of the high ionic conductivity of the gels in combination with their excellent adhesion to the mostly anionic, silicate-rich concrete surface [19]. Despite these advancements, highly alkaline ionic hydrogels continue to face significant challenges, particularly under extreme pH conditions. Firstly, the monomer chains are susceptible to Hofmann-like degradation. This issue can be mitigated by modifying the chemical structure of the tertiary nitrogen-containing monomer units, thereby enhancing the overall alkali stability of the hydrogel [20]. Secondly, the long-term effectiveness of these hydrogels is critically dependent on the stability of the crosslinking. Recent studies have shown that the use of tetraallyl crosslinkers, such as tetraallylammonium bromide (TAAB) and tetraallyltrimethylene dipiperidine dibromide (TAMPB), not only improves alkali resistance but also ensures a more uniform distribution of crosslinks compared to conventional crosslinkers like bismethacrylamide (BIS) [21,22].

However, two primary challenges remain unresolved: the presence of halide ions (chloride or bromide) in these new crosslinkers, which raises concerns, particularly for applications involving steel-reinforced structures, and the ongoing difficulty of efficiently producing large quantities of highly alkaline DADMAOH solutions. Current production methods rely on ion-exchange resins, which are not ideal in terms of time and energy efficiency. These methods involve the use of exchange columns that require significant dilution, followed by a lengthy, careful, and energy-intensive concentration step [18]. To overcome these limitations, a new process was developed to convert commercially available, pH-neutral diallyldimethylammonium chloride (DADMAC) solutions directly into DADMAOH gels, with a focus on efficiently producing gel particles.

## 2. Results and Discussion

Initial attempts to make the ion-exchange process more efficient but still use ion-exchange resins were carried out using a concentrated monomer solution with a target concentration of 2 M. This is the maximum concentration at which DADMAOH can be handled without decomposition (30 wt%) [18]. Starting with and maintaining this concentration throughout the entire exchange process would save the energy-intensive and time-consuming step of concentrating the exchanged product. However, the ion-exchange efficiency of the anion-exchange resin decreased significantly at these monomer concentrations, leaving chloride contents of over 80% after the first cycle. Multiple cycles led to increasing decomposition of the monomer due to the increased exposure of the alkaline resin. 

**Figure 1:**
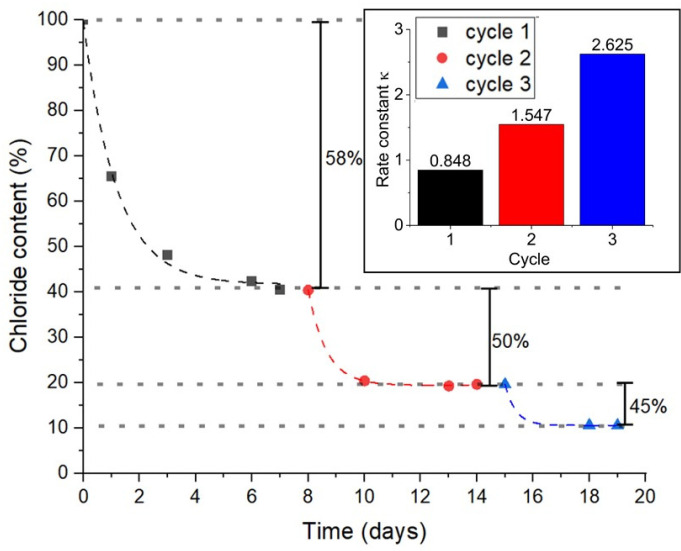
Profile of the chloride content of a 2 M DADMA-chloride/hydroxide solution over three exchange cycles against a 2 M NaOH solution separated by a cation-selective membrane, along with the corresponding rate constants κ. The NaOH solution was replaced after each cycle of 7 days.

The second attempt to replace the ion exchange resin was made by using a cation-selective membrane, clamped tightly between two differently sized polycarbonate containers, each open on one side. Such membranes are already being used in fuel cells [23]. That setup is similar to the one used here [24] but is larger in size (Figure S1). Like the DADMA^+^ monomer, the membrane consists of quaternized ammonium ions, which should prevent the passage of cations due to the density of positive charges. For the experiment, the concentrations of both DADMAC and the NaOH solution were adjusted to 2 M, as this corresponds to the desired final DADMAOH concentration and prevents the build-up of osmotic pressure. The exchange was run in three cycles of 7 days each, using fresh NaOH solution at the beginning of each cycle. The chloride content on the DADMA^+^ side was monitored by potentiometric titration over the entire period (Figure 1). 

Due to the different sizes of the chambers and the NaOH solution being in the larger one, 71.4% of the chloride anions can theoretically be exchanged in one cycle. However, Figure 1 shows that the percentage of exchanged chloride ions decreases from 58% in the first cycle to 45% in the third. The decrease in the chloride concentration over time can be described with a first-order exponential decay in all three cycles using $Y=A\cdot e^{-kt}+Y_{0}$, where *Y* is the chloride content and *Y*_0_ is the equilibrium content at the end of each cycle. The values can be found in supporting Figure S2. The rate constants for the individual cycles are depicted in the inset of Figure 1.

Over the cycles, *k* increases by a factor of approximately 3, from 0.848 to 2.625. This means not only that the percentage of exchanged chloride ions decreases but also that the time to reach equilibrium becomes shorter. Based on these changes in the process, it can be estimated that another two cycles are necessary to obtain a chloride content below 4%. The reason for this declining performance only became clear after determining the solid content of the DADMA^+^ side by lyophilizing small aliquots of the solution. It was found that the solids content decreased by approx. 20% in the first cycle and another approx. 10% in each of the following two cycles. This can be explained by the hydrolytic decomposition of the DADMA^+^ monomers, which manifests itself in a yellow discoloration of the solution and an unpleasant fishy odour. After the first cycle, the pH value on the DADMA^+^ side is already >13, and previous experiments have shown that DADMAOH solutions of comparable pH and concentration must be stored in a cool place. The decreasing rate constant of the exchange suggests that it is possible to significantly reduce the cycle time from 8 days in the first cycle to 2–3 days in the second and third cycle. This would reduce the exposure time of the monomer to the increasingly alkaline medium and, potentially, reduce monomer decomposition. However, with the discovery of an alternative, simpler method, the membrane process was abandoned.

Previous work has shown that polymerized DADMAOH gels are significantly more resistant to alkaline hydrolysis than monomer [22]. Moreover, each swollen gel particle represents an individual phase with a chemical potential that differs from the surrounding solution. The particles are also separated from the surrounding medium by a more or less clear phase boundary that is open for the exchange of dissolved mobile substances. Thus, the particles should be able to act as microsized exchange chambers similar to the experiment above. Due to the upper limit in the swelling degree, the difference in the chemical potential cannot be compensated by the uptake of water, which results in a constant driving force to exchange ions with the surroundings. This led to the idea of using the polymerized gel itself as a stationary ion exchanger. Thus, placing DADMAC particles in an excess NaOH solution generates a concentration gradient of mobile anions, facilitating a diffusion-driven exchange of chloride ions by hydroxide ions, thereby avoiding the decomposition of DADMA^+^ cations. Furthermore, the polymerization of a neutral DADMAC gel offers several advantages over the polymerization of the alkaline DADMAOH monomer: (i) the polymerization can be carried out from a commercially available, storable 65 wt% (4 M) DADMAC solution; (ii) the bromide counterions of the cationic crosslinkers such as TAAB or TAMPB are also exchanged for hydroxide ions, which reduces the residual halide content of the resulting DADMAOH particles; and (iii) the particle properties can be modified to a greater extent, as the crosslinkers and with them the resulting crosslinking densities can be varied in a significantly wider concentration range.

**Figure 2:**
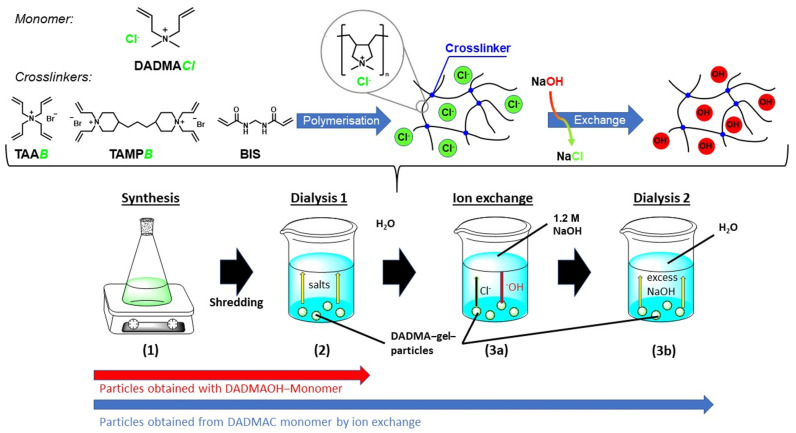
Schematic illustration of the preparation of highly alkaline DADMAOH gel particles: (1) synthesis of a DADMAC bulk gel followed by coarse shredding, (2) dialysis of the crushed DADMAC gel particles against water to remove residual salts, and (3a) ion-exchange dialysis against 1.2 M NaOH. (3b) Final dialysis against water to remove excess NaOH. The synthesis directly from DADMAOH monomer (red arrow), involves only synthesis, shredding and dialysis (1 + 2), whereas the particle synthesis from neutral DADMAC (blue arrow) includes all steps (1−3b).

Thus, DADMAOH particles are produced by first synthesizing a DADMAC bulk gel, which can easily be scaled up. After curing, the gel is vigorously stirred in an excess of water, and the resulting particle dispersion is then dialyzed in three steps. An illustration of the preparation process can be seen in Figure 2. Firstly, dialysis against deionized water is carried out to remove residual monomers and the remains of the initiator system from the gel (step 2 in Figure 2). The halide ions are then exchanged for hydroxide using a sodium hydroxide solution (step 3a in Figure 2), and, in the final step, an excess of NaOH is removed by another dialysis against deionized water (step 3b in Figure 2). Dialysis bags can be used for easier handling but are not absolutely necessary. The residual halide content of the resulting particles was analysed by potentiometric titration and was determined to be <0.3%. On the other hand, DADMAOH gels prepared from the alkaline monomer with TAAB or TAMPB as crosslinker still contain approx. 5–10% of halide ions [18].

To rule out the possibility of the gel decomposing during the ion-exchange process, the degree of swelling was analysed directly after synthesis and crude comminution in the chloride form (steps 1 in Figure 2), after dialysis in water (step 1 + 2 in Figure 2), and after ion exchange with NaOH with subsequent dialysis against water (steps 1–3b in Figure 2). For this purpose, gels were prepared from a 2 M or a 4 M DADMAC solution following the procedure in Figure 2 with 20 mol% (based on the amount of DADMAC) tetraallylammonium bromide (TAAB) or 15 mol% *N,N,N*’,*N*’-tetraallyltrimethylene dipiperidine dibromide (TAMPB) as crosslinker. The difference in the crosslinker concentration is due to the solubility of the crosslinkers and the better handling of the more highly crosslinked TAAB gels. A detailed discussion of the influence of the different crosslinkers and crosslinker content can be found in a previous article [21]. In summary, the use of TAAB allows for the production of gels with higher swelling capacities compared to those crosslinked with TAMPB, while also being significantly easier and more cost-effective to manufacture. High degrees of swelling are particularly beneficial for applications such as the realkalization of carbonated concrete, as they enable the system to retain more water, which can then serve as a medium for ion transport within the pore structure. Additionally, increased swelling properties are advantageous for sealing water-bearing cracks in structures. By contrast, gels crosslinked with TAMPB exhibit significantly lower degrees of swelling, which is critical for use in cementitious materials. Excessive volume changes and the resulting swelling pressure could lead to spalling, making lower degrees of swelling preferable for these applications. The 2 M DADMAC solution was chosen as this corresponds to the maximum concentration of the DADMAOH solution not showing decomposition. The 4 M DADMAC solution is the commercially available form and was included to check for potential concentration effects. Gels prepared from 2 M DADMAOH with both crosslinkers were used as reference. As these do not require the ion-exchange step, they were only dialyzed once against distilled water after crude crushing (steps 1 + 2 in Figure 2).

**Figure 3:**
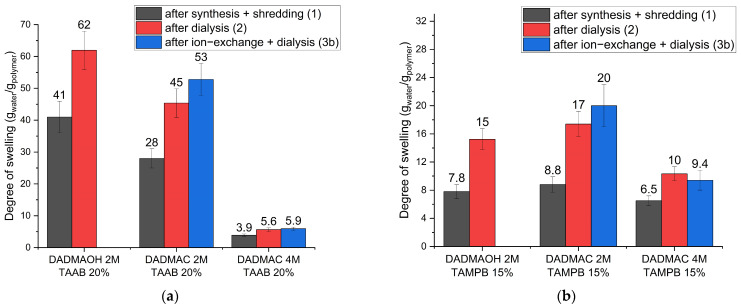
Degree of swelling of DADMA^+^ gels with 20 mol% TAAB (**a**) or 15 mol% TAMPB (**b**) as crosslinker after synthesis + shredding (black), after dialysis against doubly distilled water (red), and after ion exchange with NaOH with subsequent dialysis against water (blue). Gel particles produced directly from 2 M (30 wt%) DADMAOH (no ion exchange required) are used as reference. Please note the different scaling of the Y-axes.

It can be observed that the degree of swelling of all samples increases after the first dialysis in water (Figure 3 black to red) by a factor of approx. 1.6 ± 0.16. This is likely due to the presence of residual monomers and initiator salts in the samples immediately after synthesis. These charged species contribute to an elevated ionic strength within the gel and an increase in the content of solids, both of which adversely impact the swelling capacity. For TAAB-crosslinked gel particles, it is evident that particles prepared from 2 M DADMAC solution (Figure 3a, centre) show lower degrees of swelling than those prepared from DADMAOH (Figure 3a, left) at the same concentration. This is not observed with TAMPB as crosslinker (Figure 3b left and centre). A similar trend has previously been observed for DADMAC and DADMAOH bulk gels [21,22]. It seems that TAAB crosslinks less efficiently in alkaline environments, thereby producing a less dense network, which exhibits a higher degree of swelling. A potential explanation is that hydroxide ions are less effective at shielding positive charges compared to chloride ions, which further impedes the already challenging second ring closure of TAAB, as the positively charged polymer chains must come into close proximity with the relatively small crosslinker. This effect is not observed with TAMPB, where the larger molecular size allows for greater inter-chain distances and the crosslinking reaction proceeds more efficiently.

The degree of swelling increases slightly further after ion exchange and final dialysis against water (Figure 3, blue), except for the gel prepared from 4 M DADMAC, although the confidence intervals overlap in all cases, so this cannot be decided with final certainty. The reduced shielding effect of hydroxide ions again seems to be a key factor. Upon replacing chloride with hydroxide, the Debye length of the positive charges along the polymer chains increases, leading to a moderate increase in the degree of swelling. This effect is more pronounced in the less crosslinked samples prepared from 2 M DADMAC solutions. In TAAB-crosslinked samples, the swelling follows the trend 2 M DADMAOH > 2 M DADMAC, as the shielding effect appears to be insufficient to counterbalance the lower crosslinking density of the DADMAOH particles (vide supra). In TAMPB-crosslinked samples prepared from 2 M solutions, the values are similar when taking the confidence interval into consideration. Since TAMPB was not found to show different crosslinking efficiencies at different pH levels and the particles synthesized from DADMAOH contain approx. 30% of halide counterions, this observation corroborates the hypothesis that the influence of crosslinking outweighs shielding in terms of swelling.

**Figure 4:**
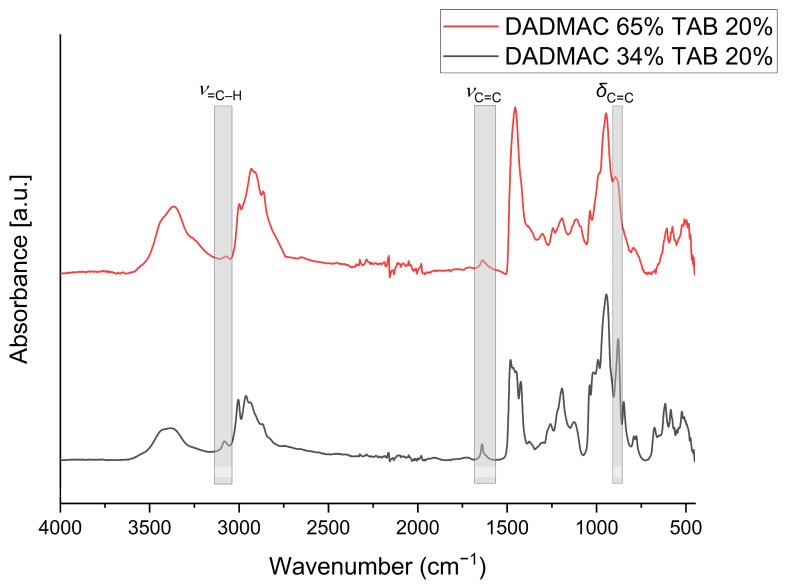
FT-IR spectra of dried gel particles prepared from a monomer solution with 4 M (65 wt%) (upper line) or 2 M (34 wt%) DADMAC (lower line) crosslinked with 20 mol% TAAB. The grey areas mark the characteristic allyl vibrations.

From a broader perspective, in the set of gels prepared from 4 M solutions, the TAAB-crosslinked gels (Figure 3a, right) exhibit lower degrees of swelling compared to the TAMPB-crosslinked gels (Figure 3b, right). This aligns with the higher calculated crosslinker content but is contrary to the behaviour of gels prepared from 2 M solutions (Figure 3, centre columns). As previously reported, TAAB is often only incorporated as a linear segment using only two of its allyl groups, as the second ring closure would form a 4,4-spiro compound with increased ring strain [21]. The actual crosslinking density of TAAB-crosslinked gels is, therefore, lower than the calculated one. TAMPB has a greater distance between the polymerization sites and does form 4,5-spiro compounds, whose ring tension is significantly lower [21]. The results observed for the gels prepared from 4 M solutions suggest that TAAB is more efficient at crosslinking in concentrated monomer solutions, which is consistent with the trend seen in bulk gels. To corroborate this, polymerization mixtures were prepared from 2 M and 4 M DADMAC solutions both containing 20 mol% TAAB based on the amount of DADMAC. This resulted in the mixtures having a crosslinker concentration of 0.8 M and 0.4 M, respectively. The solutions were cured, and the resulting gel samples were dried for analysis by infrared spectrometry (Figure 4).

**Figure 5:**
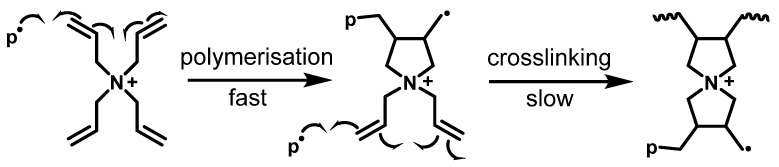
Ring-closing reaction of the polymerization of TAA^+^. P^·^ represents the attacking polymer radical, and the twist lines indicate the continuation of the polymer chain.

Focusing on the specific allyl vibrations, the intensities of δ_C=C_ at 922 cm^−1^, ν_C=C_ at 1647 cm^−1^, and ν_=C–H_ at 3079 cm^−1^ are significantly lower for gels made from 4 M DADMAC (Figure 4, upper line) than for gel particles made from 2 M DADMAC (Figure 4, lower line). Both gels were dialyzed before the analysis to remove unreacted monomer and initiator salts so that the observed allyl vibrations could only originate from partially polymerized TAAB. As the ratios of monomer to crosslinker were kept identical, the lower allyl intensities in the spectrum of the 4 M gel clearly indicate that the TAAB crosslinker reacted more frequently on both sides and thus gives rise to higher crosslinking densities. This is in line with the results in Figure 3a, where a lower degree of swelling was observed for gels prepared from 4 M solutions. This explanation is supplemented by the concentration-related proximity of the cationic molecules [21]. It is known that pure DADMAC solutions polymerise faster with increasing concentration, as the distances between the repelling cationic monomers become smaller [25]. A similar behaviour can therefore be assumed for the TAAB crosslinker due to the structural similarity to DADMAC so that the second polymerization step for crosslinking is facilitated by the smaller distances within the solution compared to less concentrated solutions, as shown in Figure 5. Given the observed concentration-related effect of TAAB crosslinking, it may be possible to adjust the degree of swelling by simply varying the DADMAC concentration, in addition to the traditional approach of varying the crosslinker content. Polymerization from DADMAOH also further limits the degree of swelling that can be achieved, as an increased addition of TAAB of >30 mol% promotes the hydrolysis of the DADMAOH-monomer solution by reducing the distance between the monomers.

The DADMA^+^ particles produced from 4 M DADMAC were crushed using a dissolver disc and Ultraturrax before the ion-exchange step, and the particle diameter was determined by light microscopy to be approx. 140 µm on average. During dialysis, agglomeration was observed, which necessitated an additional shredding step after ion-exchange dialysis (step 3b in Figure 2). The resulting particle size distribution was then reanalysed. The corresponding microscopy images can be found in the Supporting Information Figure S3. Due to the shredding method, the particles are not round but irregular with a shape factor of 0.16 ± 0.12. The particle diameters were measured automatically and determined to be 19.56 ± 15.98 µm. The second shredding process therefore reduced the average particle size by a factor of approximately seven. This is particularly important for applications in cementitious building materials, as the ratio of the particle radii to the average size of the capillary pores of mortar or concrete has a massive influence on the corresponding strength of the resulting components when used, e.g., as a shrinkage reducing agent.

**Figure 6:**
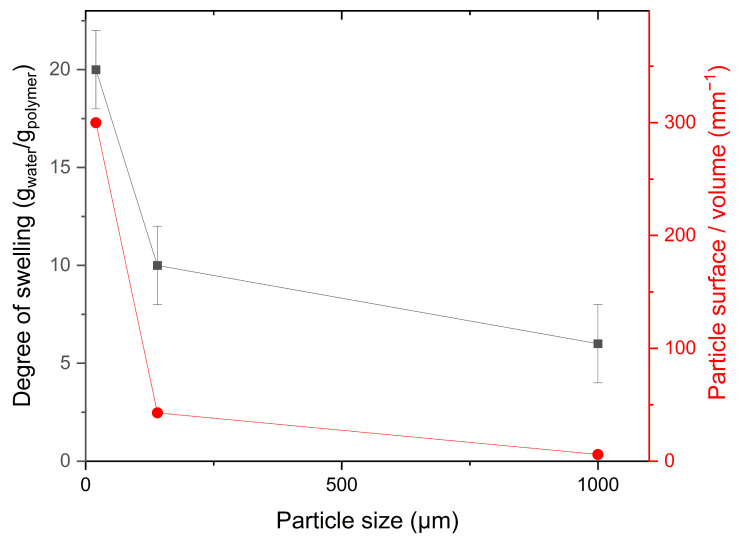
Degrees of swelling of DADMAOH-*co*-TAAB gels (made from 4 M DADMAC and 20 mol% TAAB) in double-distilled water in relation to the average particle diameter (black squares). The red dots show the calculated surface-to-volume ratio of the particles against the particle diameters assuming a spherical geometry.

It was observed that the degree of swelling of the resulting DADMAOH gel particles increases with increasing size reduction. Therefore, DADMAOH-*co*-TAAB particle samples were analysed for their swelling capacity at different stages of comminution. The corresponding values are shown in Figure 6.

Particles with only coarse comminution and a diameter of approx. 1 mm showed a water uptake of 6 g_water_/g_dry polymer_, which corresponds to the value of the continuous network. After the first comminution step, but before the ion exchange (step 2 in Figure 2), the particles had a diameter of approx. 140 µm and took up 10 g/g, while after the second comminution step, the diameter decreased to approx. 20 µm and the uptake rose to approx. 20 g/g (see Figure 6, black dots). The course of the degree of swelling as a function of the particle diameter seems to follow an exponential curve, and it turned out that its shape roughly matches the surface-to-volume ratio of spherical particles (Figure 6, red dots). Polymer charges on the surface of the particles can bind considerably more water than internal ones, and as the total surface area increases through fragmentation, the particles are able to retain more water per gram of polymer. A change in the degree of swelling as a function of the particle size has already been observed in the literature for polyacrylamide hydrogel particles [26]. Yan Bao et al. explained a similar observation for the water retention of cellulose-*co*-PAA superabsorbent polymers with the increase in surface area and the associated increase in contact area with water [27].

A practical application for superabsorbents in concrete is the self-healing of water-bearing cracks [28]. For this purpose, hydrogel particles are mixed in and then swell on contact with penetrating water. The particles used for this are typically in the range of 10 and 500 µm with swelling degrees between 10 and 30 times their dry weight. Both are in good agreement with the DADMAOH particles reported here. To demonstrate their general suitability, UV-active particles were synthesized by copolymerizing 4 M DADMAC with 1 mol% fluorescein-*O*-acrylate and incorporated into a porous mortar matrix. In Figure S4a, the particles are clearly visible as yellow glowing dots in the pore spaces after the preparation of the sample. The test specimens were then placed in a water bath for 28 d and then examined again. It can be seen that the particles swelled on contact with water and completely fill the pore spaces but did not migrate and were not washed out in the sample (Figure S4b). This confirms the excellent adhesion of the polycationic polymer network to the negatively charged, silica-rich surface of the concrete.

**Figure 7:**
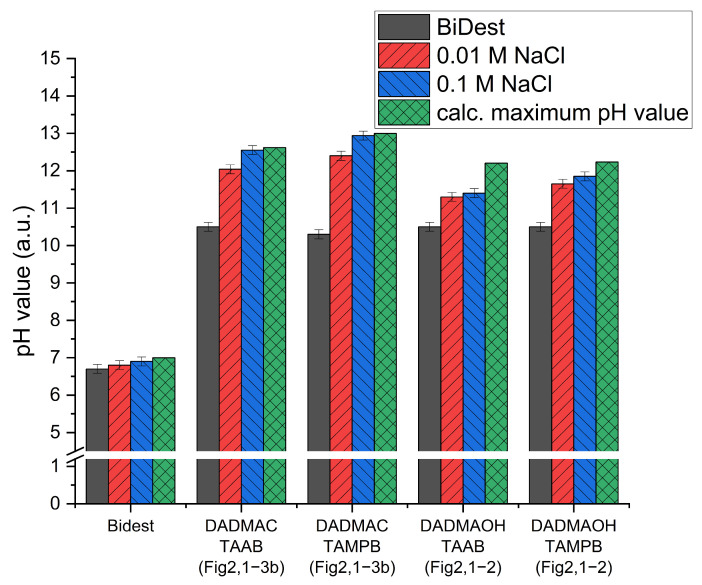
pH values of DADMAOH-*co*-TAAB/TAMPB gel particles in aqueous dispersion (approx. 2.4 g_polymer_/L) in comparison with particles prepared conventionally from 30 wt% DADMAOH solution (Figure 2, steps 1–2) and subsequently alkalized particles from a 65 wt% DADMAC solution (Figure 2, steps 1–3b). Each case is compared to its maximum values, calculated on the basis of the polymer and charge quantity.

The most important property of the particles for possible later applications in the construction industry is the alkalinity, as well as the release and exchange of the mobile hydroxide ions into the surrounding medium. To determine whether the manufacturing process has an influence on this property, particles prepared directly from 2 M DADMAOH solution and from 4 M DADMAC solution by ion exchange (Figure 2, step 1–3b) were dispersed in double-distilled water and NaCl solution, and the resulting pH value was measured. Sodium chloride was added stepwise to the dispersions to facilitate ion transport from the particles to the pH electrode (see Figure 7).

Firstly, it is notable that a pH value of approximately 10 is observed for all alkaline particles in water, which is significantly higher than the pH of pure double-distilled water, of approx. 6.7. This is surprising given that hydroxide ions should not be able to dissolve freely in the double-distilled water due to the absence of mobile cations. The higher pH can be attributed to residual NaOH, which remains in trace amounts even after triple dialysis of the particles. To confirm this, the pH values of the dialysis water were measured after each exchange, revealing that even after the third dialysis, a slightly elevated pH of around 8–9 was still detectable. In this experimental setup, only a relatively low residual concentration of 0.1 mM NaOH is needed to achieve the observed initial pH of 10. In the case of minimal environmental release during potential applications, significant contamination is unlikely, as the residual NaOH content remains too dilute to cause substantially elevated pH levels. While natural waters contain dissolved ions capable of exchanging with hydroxide ions, their concentrations are typically very low, leading to a gradual and limited release of hydroxide ions over time. Additionally, the particles are engineered for compatibility with inherently alkaline cementitious materials, whose silica-rich, negatively charged surfaces exhibit a strong affinity for the polycationic gel network, thereby enhancing binding interactions. Furthermore, for applications within the cement matrix, it has been demonstrated that the particles are not prone to leaching. For surface applications, such as electrochemical chloride extraction (ECE), physical coverings like plastic films are used to retain the particles.

The pH values of the ion-exchanged particles (Figure 7, DADMAC TAAB/DADMAC TAMPB) align closely with the calculated maximum values at the given concentrations, which are based on the amount of DADMA^+^ units. By contrast, particles produced directly from a 2 M DADMAOH solution (Figure 2, steps 1–2) do not reach their calculated target pH values, regardless of whether TAAB or TAMPB is used as a crosslinker. This discrepancy is likely attributed to incomplete ion exchange within the monomer solution, which retains up to 10% residual content, as well as the use of crosslinkers in their bromide form (ranging from 1–30 mol% relative to monomer content), resulting in a considerable residual halide content (vide supra). Conversely, gel particles synthesized from DADMAC (Figure 2, steps 1–3b) exhibit residual halide contents of less than 0.3%, as confirmed by potentiometric titration with silver nitrate. This observation extends to the crosslinking agents; in particles synthesized directly from DADMAOH, the crosslinkers remain in the bromide form. However, following subsequent ion exchange, the crosslinkers in the processed particles are effectively converted to their hydroxide forms. Thus, it appears that chloride ions are fully exchanged for hydroxide ions during the dialysis process.

**Figure 8:**
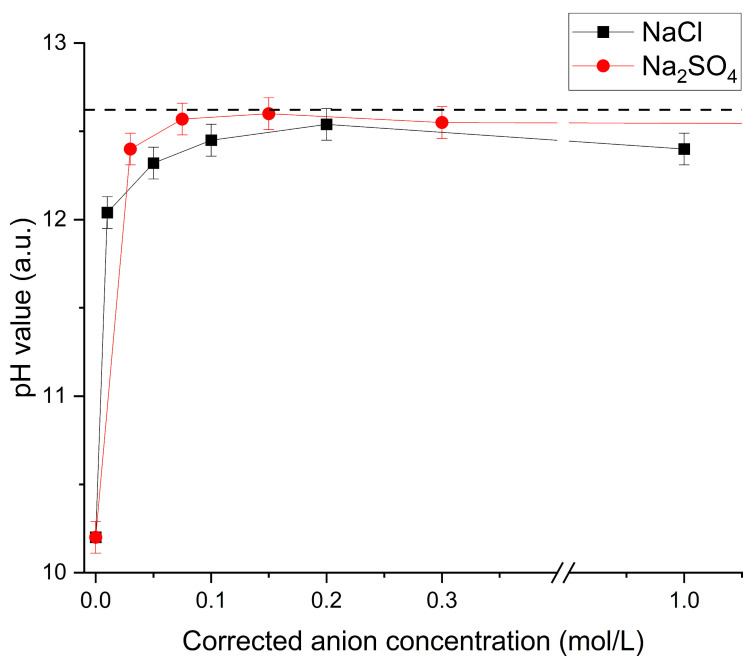
pH value of a dispersion of DADMAOH-*co*-TAAB particles made by ion exchange upon adding increasing amounts of NaCl or Na_2_SO_4_ as a function of the corrected anion concentration = *c*_i _· *z*_i_. The dashed line is the nominal pH value calculated based on the amount of polymer and charge.

This raises the question of whether divalent anions such as, e.g., sulfate trigger a different release profile of the particle-bound hydroxide ions than monovalent ions such as chloride. Figure 8 shows this comparison using DADMAOH-*co*-TAAB particles made by ion exchange. 

Mathematically, the particles can release a hydroxide concentration of 0.05 M under the conditions used, and therefore, the dispersion can reach a maximum pH value of approx. 12.62. For both sulfate and chloride, the pH value rises sharply at very low concentrations. Sulfate seems to be slightly more effective in the exchange for hydroxide at low concentrations, expelling all hydroxide anions at the calculated charge concentration of 0.05 M (pH = 12.6), while approx. twice this amount is necessary with chloride (pH = 12.55). Note that charge concentration refers to the molar amount of anions *c*_i_ multiplied by its charge *z*_i_. In previous studies, it was observed by infrared spectroscopy that the resonance stabilization of carbonate decreases upon absorption in an alkaline DADMAOH gel [18]. One possible explanation for this could be the bidentate charge–charge interaction of carbonate ions and the flexible polycationic network so that bidentate anions are favoured over monodentate hydroxide. A similar line of thought can be envisioned for sulfate ions, which would explain the faster release of hydroxide ions compared to NaCl, which seems to be based not only on statistical diffusion. 

Alkaline DADMAOH bulk gels crosslinked with methylene-*bis*-methacrylamide (BIS) have previously been used, e.g., as coupling material for electrochemical chloride removal [19]. Due to the low solubility of the BIS crosslinker, these DADMAOH gels require the addition of methacrylamide (MAA) as a comonomer in order to achieve the required rheological properties and reproducible, reliable gelling through additional physical crosslinking [18]. To act as a coupling material between an electrode and the concrete surface, both the adhesion to the surface and the electrical conductivity are decisive. In the bulk gel, the charge carriers can migrate directly along the polycationic polymer framework. For particulate systems, however, there are many particle boundaries to cross, which can potentially impair the conductivity. Therefore, the ion-conducting properties of the particles were analysed in direct comparison with the bulk samples, considering the different crosslinking systems. For this purpose, the following solutions were directly polymerized to bulk hydrogels inside cuvettes with integrated electrodes: 2 M DADMAOH, 2 M and 4 M DADMAC, both in the chloride form and using BIS/MAA and TAAB as crosslinkers with all three solutions. In addition, particle dispersions were prepared from these six combinations as described above, centrifuged, and injected into the measuring cuvettes. Finally, DADMAOH particles were prepared from a 4 M DADMAC solution by ion exchange (step 3b in Figure 2), centrifuged, and transferred to the cuvettes. The electrochemical impedance measurement showed a strong capacitive resistance behaviour in the low-frequency range for both the bulk gels and the particles. In all cases, however, the measured values approached the true conductivity of the solution as the frequency approached infinity. At a frequency of approx. 10 kHz, the conductivity was found to be constant for all samples, so the specific resistances at this frequency were compared with each other in the following.

**Figure 9:**
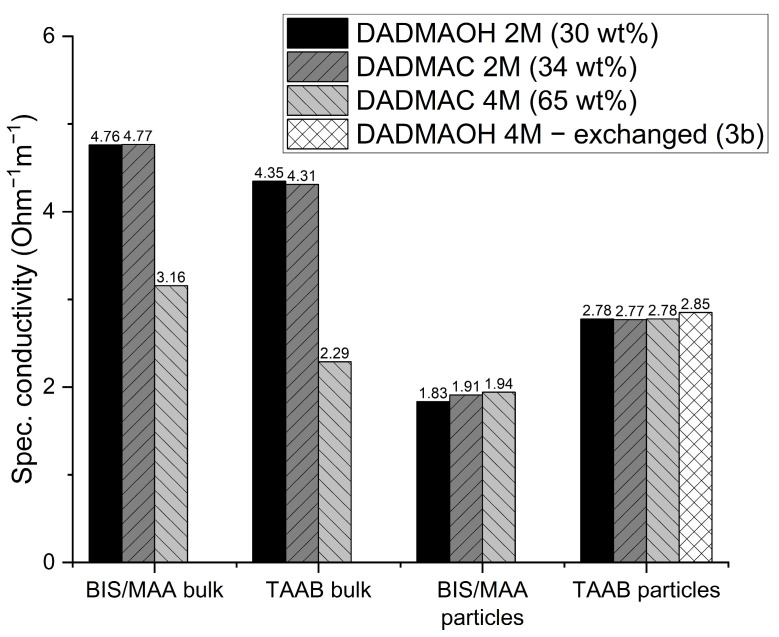
Specific conductivities at 10 kHz of DADMA^+^ hydrogels (Figure 2, Step 1) and DADMA^+^ hydrogel particle dispersions (Figure 2, Step 2) crosslinked with 4 mol% BIS + 10 mol% MAA or 20 mol% TAAB after shredding as determined by impedance spectroscopy.

The bulk gels prepared from 2 M solutions show rather similar specific conductivities independently of the type of monomer or crosslinker (Figure 9). This is remarkable as the limiting molar conductivities of OH^−^ and Cl^−^ differ by a factor of approx. 2.5 [29]. This indicates that the viscosity limits the transport rather than the intrinsic ionic mobility. For both types of crosslinking, the specific conductivities in the 4 M DADMAC (65 wt%) bulk gels are approx. 40% lower than those of the 2 M bulk gels. As shown above, the more concentrated solutions give rise to much denser networks, which also increases the viscosity inside the gels. It reduces the ion mobility and, thus, the ionic conductivity. This effect appears to outweigh the influence of the higher amount of charge carriers in the cuvette compared to the 2 M bulk gels. A comparison of the BIS/MAA crosslinked bulk gels with the TAAB crosslinked ones shows that the latter are slightly less conductive. This can be explained by the higher crosslinking density and, therefore, higher viscosity of the TAAB samples.

The situation is completely different at the particle level. For both crosslinkers, the solution concentration and, with it, the viscosity inside the particles do not affect the specific conductivity. This can be explained by the fact that, due to the large surface area after shredding, charge transport occurs primarily along the surfaces of the particles, making the viscosity effect less significant compared to that for the bulk gels. Further evidence supporting this assumption is provided by the reversal of the relationship between the two crosslinking systems. In the bulk form, where charge transport occurs only through the gel, the conductivity of BIS/MAA-crosslinked gels is higher than that of the more highly crosslinked and, therefore, more viscous TAAB-crosslinked gels. However, in particle form, the specific conductivity of the TAAB-containing gels is approximately 30% higher than that of the BIS/MAA gels. Unlike TAAB, both BIS and MAA are uncharged molecules that do not contribute to charge transport but account for 15.2% of the repeat units.

**Figure 10:**
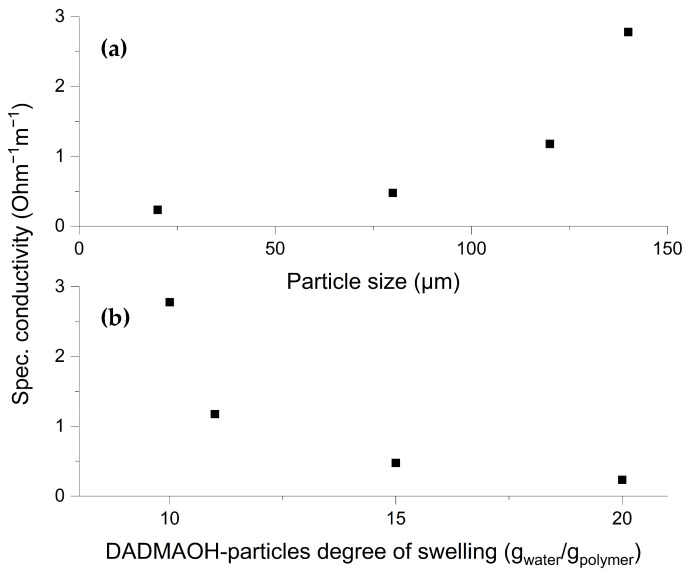
Specific conductivities of DADMAOH-*co*-TAAB hydrogel particles produced from a 4 M DADMAC (65 wt%) solution (black dots) as a function of particle size (**a**) and the degree of swelling (**b**).

It is shown in Figure 6 that reducing the particle size increases the degree of swelling. This influences the viscosity, the density of the charge carriers, and the average solids content. All these factors affect the conductivity in opposite ways, so it is interesting to see how the particle size correlates with the conductivity. To test this, DADMAOH particles were produced from a 4 M (65 wt%) DADMAC solution (step 3b in Figure 2) and then shredded to different particle sizes (Figure 10). It can be seen that the specific conductivity decreases with decreasing particle size and increasing degree of swelling. Considering the results from Figure 6, these two representations are consistent. It was concluded from Figure 6 that the increasing amount of water is mainly located at the particle surface and from Figure 9 that charge transport occurs mainly along the surface of the particles. Combining these two findings leads to the conclusion that reducing the particle size reduces the concentration of charge carriers on the particle surface, and since the conductivity is directly proportional to the ion concentration, this leads to a reduction of the conductivity. As previously shown, a small amount of NaOH remains in the particles even after dialysis and is gradually released into the surrounding solution. Consequently, with smaller particle sizes and a lower average solids content, the specific conductivity approaches that of a NaOH solution at pH 9, which is approx. 0.25 Ω^−1^ m^−1^.

## 3. Conclusions

The attempt to scale up the preparation of diallyldimethylammonium hydroxide (DADMAOH) using a cation-selective membrane was unsuccessful due to the slow process, which led to monomer decomposition. Potential improvements could include lowering the temperature or increasing the surface-to-volume ratio of the reactor.

As an innovative alternative, this study has developed a streamlined and scalable process for synthesizing highly alkaline DADMAOH particles. This method begins with neutral diallyldimethylammonium chloride gels, which can be readily produced in bulk quantities. By mechanically shredding the neutral bulk gel and suspending the resulting particles in a NaOH solution, the process exploits two distinct features of these gel particles: the fixed positive charges within the polymer network restrict mobility to anions, while the gels’ limited swelling capacity ensures that ion exchange is driven by the concentration gradient between the particles and the surrounding medium. Consequently, this approach yields DADMAOH particles with residual chloride contents of less than 0.3%, which is 30 to 100 times lower than those reported in previously established syntheses directly from DADMAOH monomers, depending on the choice of crosslinker. Moreover, this method allows for greater versatility in tuning crosslinking densities and solid contents, thus offering significant advantages for a broad range of potential applications.

A key finding of this study is the influence of size on the properties of the gel particles. Smaller particles increase the surface-to-volume ratio, enhancing water uptake in aqueous environments, which in turn affects the ionic conductivity. Although increased swelling results in lower specific conductivity, the particles still demonstrate high conductivity levels sufficient for practical applications, such as electrical coupling materials in construction. Specifically, the conductivity of the DADMAOH particles approaches 0.25 Ω^−1^ m^−1^, more than ten times higher than that of water-saturated concrete (~0.02 Ω^−1^ m^−1^). This notable conductivity makes these gels suitable for cathodic corrosion protection (CCP) of steel-reinforced concrete structures and electrochemical chloride extraction (ECE).

Overall, this work presents a novel, scalable, and more efficient method for producing DADMAOH particles with tailored properties, potentially paving the way for broader adoption in various industrial applications where high alkalinity and conductivity are required.

## 4. Materials and Methods

### 4.1. Materials

Diallyldimethylammonium chloride (65 wt% in H_2_O) and triallylamine (99%) were provided by Sigma Aldrich (Darmstadt, Germany). The anion-exchange resin Lewatit Monoplus MP 800 was obtained from Lanxess (Leverkusen, Germany). Chloroform (≥99%), double-distilled water, acetone (≥99%), sodium metabisulphite, *N,N*’-methylenebisacrylamide, potassium persulfate, potassium hydroxide, and sodium hydroxide (97%) were bought from VWR International GmbH (Darmstadt, Germany). 1,3-bis (4-piperidyl) propane (97+%), allyl bromide (99%) and potassium carbonate (99%) were purchased from Alfa Aesar (Kandel, Germany). The polyester filter bags with a maximum mesh size of 90 µm were obtained from Rosin Tech Products (Bethpage, NY, USA). Membra-Cel dialysis tubes with a cut-off of 14,000 Da were purchased from Carl Roth GmbH (Karlsruhe, Germany) and the cation-selective membrane Fumasep FAA-3-PK75 was supplied by FUMATECH BWT GmbH (Bietigheim-Bissingen, Germany).

### 4.2. Ion Exchange Process Using an Ion-Selective Membrane

Chambers open on one side with internal dimensions (*l* × *w* × *h*) of 10 × 10 × 10 cm^3^ and 4 × 10 × 10 cm^3^ and a wall thickness of 1 cm each were produced from polycarbonate. The front wall surfaces of the open side were covered with EPDM sealing mats, and the cation-selective membrane was sandwiched between the two cells. To ensure tightness, the setup was fixed with screw clamps. Each chamber was placed on a magnetic stirrer to ensure uniform mixing.

For the ion-exchange experiments, the smaller chamber was filled with 300 mL of a 2 M DADMAC solution, and the larger chamber was filled to the same level with 750 mL of a 2 M NaOH solution. Aliquots of 2 mL were taken from the “DADMAC side” at intervals of 1 to 2 days using a syringe and analysed for chloride content using potentiometric titration with silver nitrate. The solution was diluted and neutralized with nitric acid to prevent the precipitation of silver hydroxide. After 7 and 14 days of the experiment, the “NaOH side” was emptied using a syringe and filled with fresh 2 M NaOH solution.

### 4.3. Synthesis of the Crosslinkers

Tetraallylammonium bromide 1a (TAAB) and *N,N,N’,N*’-tetraallyltrimethylenedipiperidine dibromide 1b (TAMPB) were synthesised as described before [15]. Briefly, TAAB was prepared by reacting triallylamine with allyl bromide in acetone (80 °C, 72 h). The synthesis of 1b was performed in two steps. First, 1,3-bis (4-piperidyl) propane was reacted with 2.2 eq. allyl bromide in H_2_O (20 °C, 48 h). The purification resulted in crude diallyltrimethylenedipiperidine (DAMP) as a brown oily liquid. For the second step, the liquid was heated with 2.2 eq. allyl bromide in acetone (40 °C, 72 h) to give TAMPB as a beige crystalline solid.

### 4.4. Preparation of Diallyldimethylammonium Hydroxide (DADMAOH) by Ion Exchange

A total of 1125 g of the ion-exchange resin in the chloride form and 1.8 L of 1 M NaOH were placed in a chromatography column. The mixture was allowed to sit for 30 min before the column was drained and the resin washed with 1.2 L bidistilled water. For the exchange, 360 g of the 65 wt% commercial diallyldimethylammonium chloride (DADMAC) solution were diluted with 750 mL water and then slowly fed onto the column followed by 2.25 L double-distilled water. The combined solutions were adjusted to a concentration of 30 wt% by rotary evaporation [18]. The dry weight was determined gravimetrically by freeze-drying 5 mL of the solution. The amount of chloride left in the product was determined by potentiometric titration and was for all samples less than 10 mol%.

### 4.5. Synthesis of Crosslinked DADMAOH-Hydrogels and DADMAOH-Hydrogel-Particles 

The method to synthesise highly alkaline DADMAOH-particles is described by way of example starting from 65 wt% DADMAC solution and using 20 mol% of TAAB as crosslinker. For DADMAOH bulk hydrogels, a 30 wt% DADMAOH solution (from Section 4.4) was used and the dialysis steps against NaOH solution described below were omitted. Details on all compositions used can be found in Table S1 in the Supporting Information. A mixture of 5 g of a DADMAC solution (65 wt% in water, 20 mmol), 30 mg sodium disulfite (0.16 mmol) and 108 mg tetraallylammonium bromide (4 mmol, 20 mol% relative to the DADMAC content) was stirred until the crosslinker had completely dissolved. Meanwhile, 60 mg KPS were dissolved separately in 1.5 mL H_2_O and then added to the monomer solution. All solutions were stirred for 10 min and then stored at room temperature for 2 weeks. After curing, the gels were completely swollen in an excess of deionized water and crushed coarsely for 20 min at 700 rpm using a dissolver agitator R1300 (IKA-Werke GmbH & CO. KG, Staufen, Germany). The particles were then dispersed under ice-cooling for 15 min with a T 18 digital Ultra-Turrax (IKA-Werke GmbH & CO. KG, Staufen, Germany) at 15,000 rpm, and the resulting dispersion was dialyzed 3 times against deionized water to remove leftover monomer and salts. The dialysis tube with the particles was then dialyzed 3 times against a 1.2 M NaOH solution and then again 3 times against deionized water. When starting from a DADMAOH solution, the dialysis in NaOH solution and water is not necessary. Since the particles agglomerate slightly during dialysis, the final mixture was again treated with an Ultra-Turrax for 15 min under ice-cooling and centrifuged for 10 min at 7000 rpm to remove excess water. 

### 4.6. Swelling Experiments (Teabag Tests)

All tested hydrogel particles were lyophilized in a thin layer on the edge of a flask under reduced pressure to avoid decomposition as a result of slow drying. Approx. 100 mg of the dried hydrogels were weighed into polyester filter bags and submerged in 0.5 L of double-distilled water at 20 °C. Every hour, the filter bags were removed from the solution and carefully stripped off. To drain unabsorbed water, the samples were hung up for 5 min before the mass changes were measured gravimetrically. The maximum swelling ratios were determined by averaging the last three recorded values after weight constancy was established in the measured values. As an example, the plot of a time-dependent swelling in double-distilled water is provided in the Supporting Information, Figure S5.

### 4.7. Infrared Spectroscopy (FTIR-ATR)

The infrared spectra were recorded using a Perkin Elmer Spectrum Two UATR FTIR spectrometer from PerkinElmer Inc. (Waltham, MA, USA), equipped with a diamond ATR (attenuated total reflection) window. All spectra were recorded in the spectral range of 4000–400 cm^−1^ with 12 scans at a spectral resolution of 4 cm^−1^. Before each measurement, the diamond ATR crystal was cleaned with isopropanol. 

### 4.8. pH Value Experiments

Gel particles crosslinked with 20% TAAB (exchanged particles TAAB) and 15% TAMPB (exchanged particles TAMPB) were prepared from a 65 wt% DADMAC solution and a 30 wt% DADMAOH solution, as described above. The respective gel particle samples were stirred into double-distilled water (BiDest), and the pH value was measured using a pH meter “edge” from Hanna instruments (Vöhringen, Germany). NaCl/Na_2_SO_4_ was then added to all samples to gradually increase the concentration to 0.01 M and 1 M, and the pH value was determined once a stable equilibrium was reached. The achievement of stable equilibrium was determined by reaching a stable pH value for >5 min. The maximum pH values were calculated based on the amount of DADMAOH, which was determined from the weight of the swollen particles using the known solids content and then divided by the molar mass of a DADMAOH monomer unit (143.23 g/mol).

### 4.9. Light Microscopy

All microscopic images were recorded on an Olympus BX53M light microscope from Olympus Corporation (Tokyo, Japan). Optical lenses with 1.25× to 10× magnification were used with incident and transmitted light. Particle sizes and distribution were measured and analysed using Olympus software PRECiV Pro 1.2 (Build 27732).

### 4.10. Electrochemical Impedance Spectroscopy (EIS)

Impedance spectra were recorded on a Gamry Potentiostat 400 (C3 Prozess und Analysetechnik GmbH, Munich, Germany). Hydrogels were prepared from 3 different monomer solutions: DADMAOH 30 wt%, DADMAC 34 wt% (equimolar with the DADMAOH solution) and DADMAC 65 wt%. For bulk gel experiments, the solutions were polymerized in standard electroevaporation cuvettes from VWR International bvba. (Leuven, Belgium) with a gap width of 4 mm, equipped with two opposing aluminium electrodes of 9 × 18 mm^2^ (blocking electrodes). In the case of measurements involving gel particles, the particles were injected into the cuvettes after synthesis. Crosslinker contents of 20 mol% TAAB/4 mol% BIS were used according to instructions in 4.5. The EIS was performed at frequencies of 0.1 to 1 × 10^6^ Hz with an AC voltage of 141 mV. The resulting resistances Z were converted into the specific resistances using the electrode geometry and, thereafter, into the specific conductivities κ as reciprocal values. An example of the frequency versus real impedance (*Z*_real_) of DADMAC is provided in the Supporting Information in Figure S6.

## Data Availability

The original contributions presented in the study are included in the article/Supplementary Material, further inquiries can be directed to the corresponding author.

## Supplementary Materials

The following are available online at https://www.mdpi.com/article/10.3390/gels10110743/s1. Figure S1: Membrane experiment for ion exchange: The left compartment was filled with 200 mL of a 4 M DADMAC solution, while the right compartment contained 2 M NaOH. The casing was made of polycarbonate and placed on a multi-magnetic stirrer at room temperature. Figure S2: Chloride content profile of a 2 M DADMA-chloride/hydroxide solution over 3 exchange cycles against a 2 M NaOH solution separated by a cation-selective membrane. The NaOH solution was replaced after each cycle of 7 days. Figure S3: Light microscopy under incident light at 10× magnification of DADMAOH65-TAB particles that had been crushed twice and subsequently centrifuged. Figure S4: UV microscopy images of a mortar matrix containing 1 wt% particle admixture. Particles were synthesized from 4 M DADMAC, 25 mol% TAAB, and 1 mol% fluorescein-o-acrylate. (a) Image taken after curing under standard conditions and (b) after 28 days of water exposure. Figure S5: Exemplary plot of time-dependent swelling in double-distilled water of hydrogels of DADMAOH 30 wt%/DADMAC 65 wt%/DADMAC 34 wt% with 20 mol% TAAB crosslinking each. Figure S6: Example of the frequency versus impedance *Z*_real_ of DADMAC 65 wt% crosslinked with 20% TAAB/4 mol% BIS measured using EIS. Table S1: Details on the sample composition used to prepare crosslinked DADMAOH hydrogels and DADMAOH-Hydrogel-particles.

## References

[1] Hoffman A.S. (2012). Hydrogels for biomedical applications. Adv. Drug Deliv. Rev..

[2] Oyen M.L. (2013). Mechanical characterisation of hydrogel materials. Int. Mater. Rev..

[3] Ahmed E.M. (2015). Hydrogel: Preparation, characterization, and applications: A review. J. Adv. Res..

[4] Ranjha N.M., Mudassir J., Akhtar N. (2008). Controlled Release of Aspirin from Crosslinked Acrylic Acid Hydrogel. J. Sol-Gel Sci. Technol..

[5] Yang Y., Zhao X., Yu J., Chen X., Wang R., Zhang M., Zhang Q., Zhang Y., Wang S., Cheng Y. (2021). Efficient drug delivery and anticancer effect of micelles based on vitamin E succinate and chitosan derivatives. Bioact. Mater..

[6] Zhang Y., Wu B.M. (2023). Current Advances in Stimuli-Responsive Hydrogels as Smart Drug Delivery Carriers. Gels.

[7] Zhang C.L., Cao F.H., Wang J.L., Yu Z.L., Ge J., Lu Y., Wang Z.H., Yu S.H. (2017). Highly Stimuli-Responsive Au Nanorods/Poly(N-isopropylacrylamide) (PNIPAM) Composite Hydrogel for Smart Switch. ACS Appl. Mater. Interfaces.

[8] Murphy S., Atala A. (2014). 3D Bioprinting of Tissues and Organs. Nat. Biotechnol..

[9] Lone S., Yoon D.H., Lee H., Cheong I.W. (2019). Gelatin–Chitosan Hydrogel Particles for Efficient Removal of Hg(II) from Wastewater. Environ. Sci. Water Res. Technol..

[10] Zhou T., Yuk H., Hu F., Chen X., Guo C.F., Zhao X. (2023). 3D Printable High-Performance Conducting Polymer Hydrogel for All-Hydrogel Bioelectronic Interfaces. Nat. Mater..

[11] Shin Y. (2024). Low-Impedance Tissue-Device Interface Using Homogeneously Conductive Hydrogels Chemically Bonded to Stretchable Bioelectronics. Sci. Adv..

[12] Zhang X., Xiang J., Hong Y., Shen L. (2022). Recent Advances in Design Strategies of Tough Hydrogels. Macromol. Rapid Commun..

[13] Alzanbaki H., Moretti M., Hauser C.A.E. (2021). Engineered Microgels-Their Manufacturing and Biomedical Applications. Micromachines.

[14] Wan J. (2012). Microfluidic-Based Synthesis of Hydrogel Particles for Cell Microencapsulation and Cell-Based Drug Delivery. Polymers.

[15] Canelas D.A., Herlihy K.P., DeSimone J.M. (2009). Top-down particle fabrication: Control of size and shape for diagnostic imaging and drug delivery. Wiley Interdiscip Rev. Nanomed. Nanobiotechnol..

[16] Al-Jabari M., Ghyadah R.A., Alokely R. (2019). Recovery of hydrogel from baby diaper wastes and its application for enhancing soil irrigation management. J. Environ. Manag..

[17] Krafcik M.J., Erk K.A. (2016). Characterization of superabsorbent poly(sodium-acrylate acrylamide) hydrogels and influence of chemical structure on internally cured mortar. Mater. Struct..

[18] Jung A., Weichold O. (2018). Preparation and characterisation of highly alkaline hydrogels for the re-alkalisation of carbonated cementitious materials. Soft Matter.

[19] Jung A., Faulhaber A., Weichold O. (2021). Alkaline hydrogels as ion-conducting coupling material for electrochemical chloride extraction. Mater. Corros. Werkst. Korros..

[20] Olsson J.S., Pham T.H., Jannasch P. (2017). Poly(N,N-diallylazacycloalkane)s for Anion-Exchange Membranes Functionalized with N-Spirocyclic Quaternary Ammonium Cations. Macromolecules.

[21] Mrohs T.B., Weichold O. (2022). Multivalent Allylammonium-Based Cross-Linkers for the Synthesis of Homogeneous, Highly Swelling Diallyldimethylammonium Chloride Hydrogels. Gels.

[22] Mrohs T.B., Weichold O. (2022). Hydrolytic Stability of Crosslinked, Highly Alkaline Diallyldimethylammonium Hydroxide Hydrogels. Gels.

[23] Roschger M., Wolf S., Billiani A., Mayer K., Hren M., Gorgieva S., Genorio B., Hacker V. (2023). Study on Commercially Available Membranes for Alkaline Direct Ethanol Fuel Cells. ACS Omega.

[24] Zhao H., Zhang Y., Gong Y., Shen H., Zhang W., Cheng C., Li P. (2024). A simple method to prepare anion exchange membrane by PVA/EVOH/MIDA for acid recovery by diffusion dialysis. Water Sci. Technol..

[25] Wandrey C., Hernández-Barajas J., Hunkeler D., Capek I., Hernfández-Barajas J., Hunkeler D., Reddinger J.L., Reynolds J.R., Wandrey C. (1999). Diallyldimethylammonium Chloride and its Polymers. Radical Polymerisation Polyelectrolytes.

[26] Alvarado A.G., Arellano M., Rabelero M., Puig J.E., Sánchez-Díaz J.C. (2015). Effect of Particle Size on the Swelling and Compression Modulus of Nanostructured Polyacrylamide Hydrogels. J. Macromol. Sci. Part A.

[27] Bao Y., Ma J., Li N. (2011). Synthesis and swelling behaviors of sodium carboxymethyl cellulose-g-poly(AA-co-AM-co-AMPS)/MMT superabsorbent hydrogel. Carbohydr. Polym..

[28] Lee H.X.D., Wong H.S., Buenfeld N.R. (2016). Self-Sealing of Cracks in Concrete Using Superabsorbent Polymers. Cem. Concr. Res..

[29] Arcis H., Plumridge J., Tremaine P.R. (2022). Limiting Conductivities of Strong Acids and Bases in D_2_O and H_2_O: Deuterium Isotope Effects on Proton Hopping over a Wide Temperature Range. J. Phys. Chem. B.

