# Supplementary material for: A Simple Preparation of Crosslinked, Highly Alkaline Diallyldimethylammonium Hydroxide Hydrogel Particles via Inverse Static Anion Exchange
Source: Gels. 2024 Nov 15;10(11):743. doi: 10.3390/gels10110743 (PMC11593379; doi:10.3390/gels10110743)
Supplement: Supplementary file 1 [file gels-10-00743-s001.zip › gels-3267732-supplementary.pdf]

Supplementary Materials:

To

A Simple Preparation of Crosslinked, Highly Alkaline Diallyldimethylammonium Hydroxide Hydrogel Particles via Inverse Static Anion Exchange.

Table S1. Details on the sample composition used to prepare crosslinked DADMAOH hydrogels and DADMAOH-Hydrogel-particles.

Monomer	wt-%/conc.	Sample	Crosslinker				Methacrylamide
			Type	ratio / mol-%	n / mmol	m / mg	n (mmol) I m (mg)
DADMAC	34wt% (2M)	TAAB20	TAAB	20	2	540	
DADMAC	34wt% (2M)	TAMPB15	TAMPB	15	1,5	836	
DADMAC	34wt% (2M)	BIS/MAA	BIS	4	0,4	64	1 mmol I 89 mg
DADMAC	65wt% (4M)	TAAB20	TAAB	20	4	1032	
DADMAC	65wt% (4M)	TAMPB15	TAMPB	15	3	1597	
DADMAC	65wt% (4M)	BIS/MAA	BIS	4	0,8	124	2 mmol I 170 mg
DADMAOH	30wt% (2M)	TAAB20	TAAB	20	2	540	
DADMAOH	30wt% (2M)	TAMPB15	TAMPB	15	1,5	836	
DADMAOH	30wt% (2M)	BIS/MAA	BIS	4	0,4	64	1 mmol I 89 mg

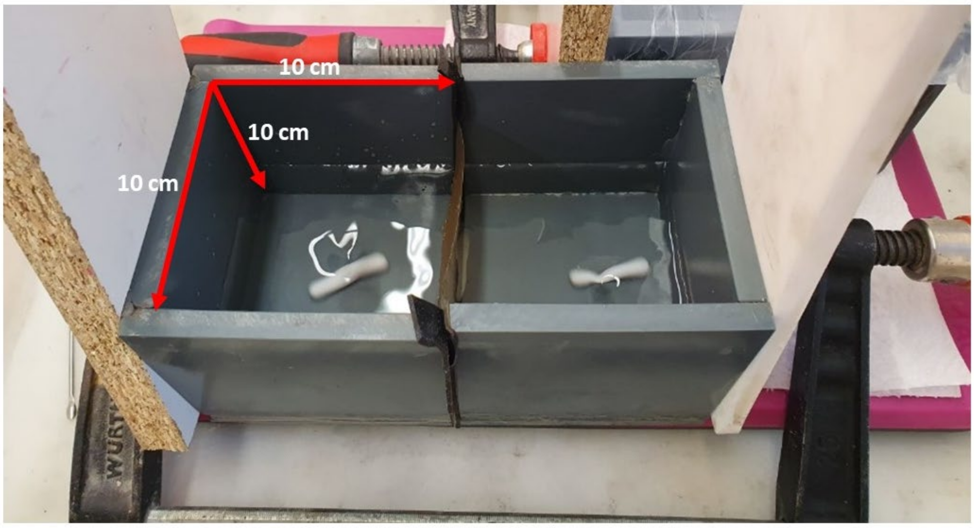

Figure S1. Membrane experiment for ion exchange: The left compartment was filled with 200 mL of a 4 M DADMAC solution, while the right compartment contained 2 M NaOH. The casing was made of polycarbonate and placed on a multi-magnetic stirrer at room temperature.

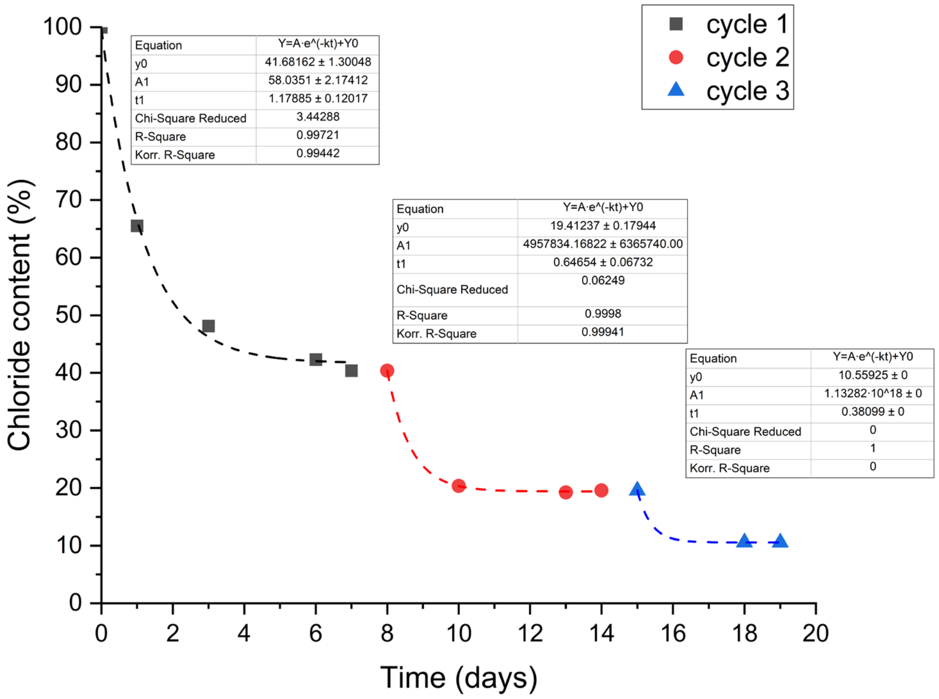

Figure S2. Chloride content profile of a 2 M DADMA-chloride/hydroxide solution over 3 exchange cycles against a 2 M NaOH solution separated by a cation-selective membrane. The NaOH solution was replaced after each cycle of 7 days.

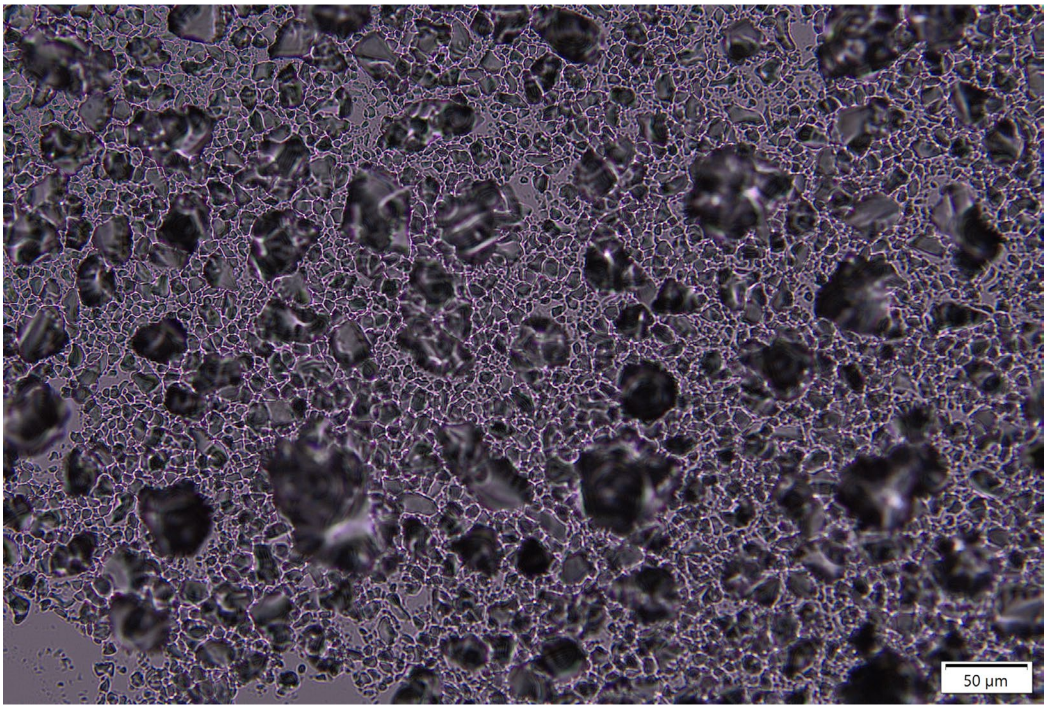

Figure S3. Light microscopy under incident light at 10× magnification of DADMAOH65-TAB particles that have been crushed twice and subsequently centrifuged.

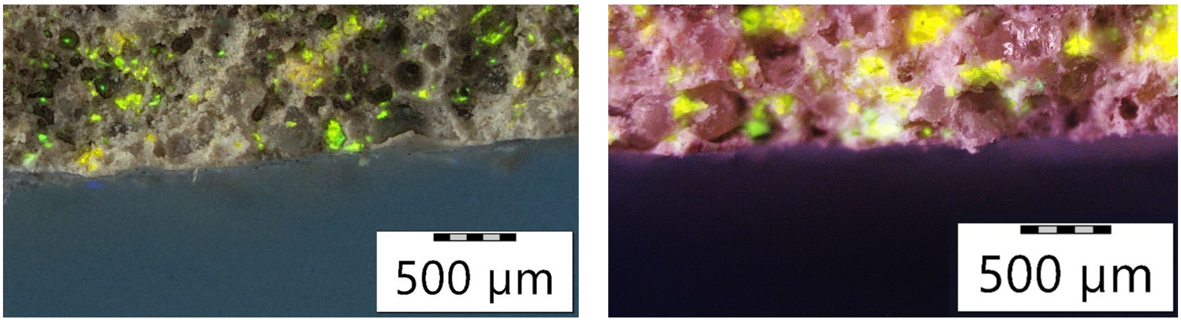

(**a**) (**b**)

Figure S4. UV microscopy images of a mortar matrix containing 1 wt% particle admixture. Particles were synthesized from 4 M DADMAC, 25 mol% TAAB, and 1 mol% fluorescein-*o*-acrylate. (a) Image taken after curing under standard conditions, and (b) after 28 days of water exposure.

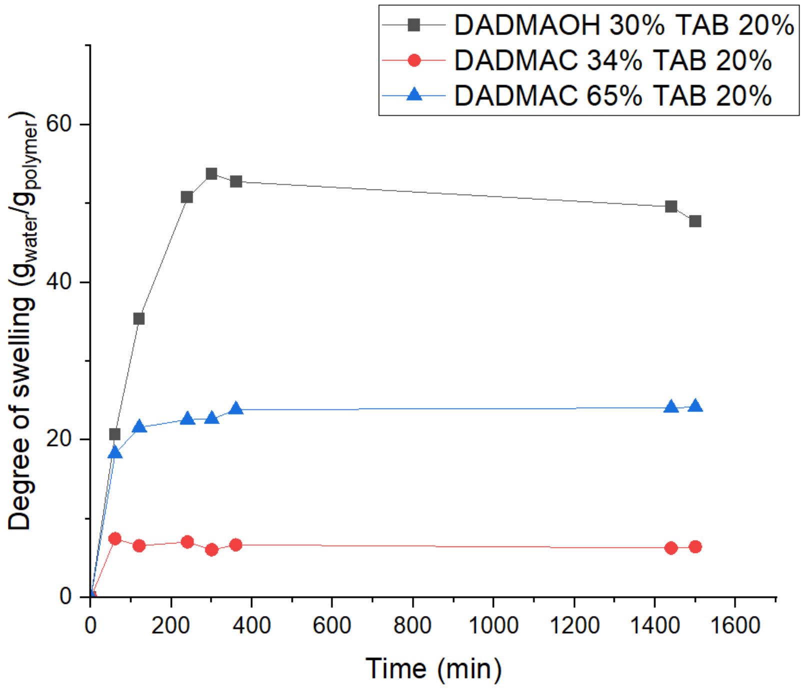

Figure S5. Exemplary plot of time-dependent swelling in doubly distilled water of hydrogels of DADMAOH 30 wt%/DADMAC 65 wt%/ DADMAC 34 wt% with 20 mol% TAAB crosslinking each.

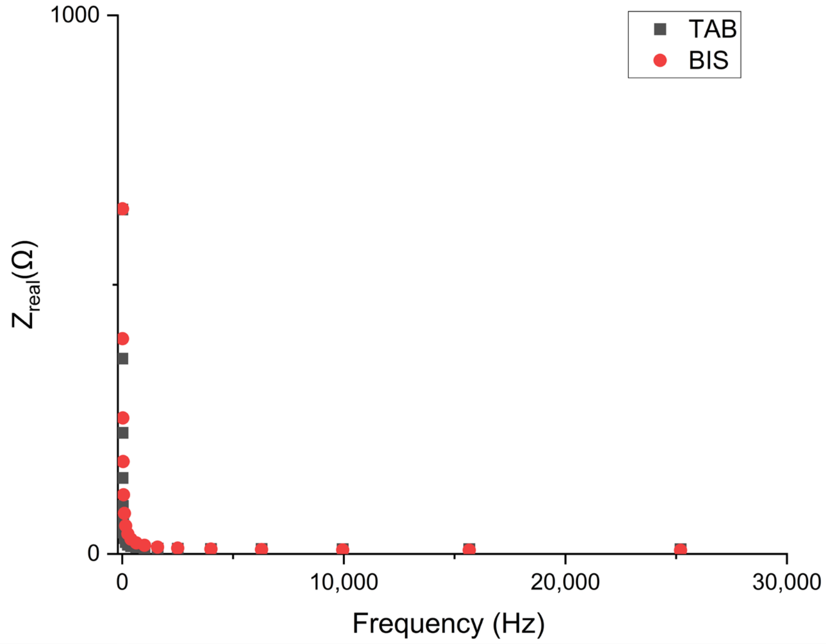

Figure S6. Example of the frequency versus impedance Z_{real} of DADMAC 65 wt% crosslinked with 20% TAAB / 4 mol% BIS measured using EIS.
